# Supplementary material for: Design method for the relocation of plastic hinges in prefabricated steel beams with corrugated webs
Source: PLoS One. 2021 Feb 19;16(2):e0246439. doi: 10.1371/journal.pone.0246439 (PMC7895350; doi:10.1371/journal.pone.0246439)
Supplement: Attachment — Submitted filename: Response to Reviewers.docx [file pone.0246439.s002.docx]

Response to Reviewers

**Response to Reviewer #1**

1.How to ensure that the component is destroyed before the node?

According to the design principle of “strong joint and weak member”， the stiffness of joints is higher than members.

2. Q235 steel, why not choose high strength steel?

The ductility of Q235 steel is better than higher strength steel.

3. The length of steel beam calculated in this paper should be greater than 2059 mm, but the actual length is 1440 mm. How to meet the shear requirements?

In addition to the beam length, the shear capacity of the beam can be improved by changing the stirrup spacing.

4. If the reinforced steel plate is welded on the flange and end plate, will the weld formed by welding cause stress concentration phenomenon on the component itself？

The weld will cause stress concentration on the component.

5. If this phenomenon occurs, should the bearing capacity and ductility of members be considered?

The effect of stress concentration is ignored.

**Response to Reviewer #2**

1.The literature review has been revised.

2.Questions 2,3,4,6 have been revised.

3. The English level should be improved by language editor.

The article has been polished by AJE.

4. In table 1, the stress-strain curves of steel should be given.

The stress-strain curve of steel is a common steel stress-strain curve, so it is not given.
